# The impact of video-animated information on anxiety, satisfaction, and pain perception in patients undergoing ESWL: a randomized controlled study

**DOI:** 10.1007/s00240-025-01757-6

**Published:** 2025-04-26

**Authors:** Recep Burak Degirmentepe, Yasir Muhammed Akca, Osman Sami Akman, Haci Ibrahim Cimen, Deniz Gul, Muammer Bozkurt, Tuncay Toprak, Osman Kose

**Affiliations:** 1 School of Medicine, Department of Urology, Sakarya University, Adapazari, Sakarya, P.C.: 54050 Turkey; 2 Department of Urology, Sakarya University Training and Research Hospital, Sakarya, Turkey; 3 Department of Urology, University of Health Sciences Prof. Dr. Cemil Tascioglu City Hospital, Istanbul, Turkey; 4 Department of Urology, University of Health Sciences, Fatih Sultan Mehmet Training and Research Hospital, Istanbul, Turkey

**Keywords:** Anxiety, ESWL, Video-animated information

## Abstract

Extracorporeal Shock Wave Lithotripsy (ESWL) is a widely used non-invasive treatment for kidney stones, but it can cause significant patient anxiety due to procedural unfamiliarity and discomfort. This study aimed to evaluate the effects of video-animated information on anxiety levels, procedural satisfaction, willingness to undergo ESWL again, and perceived pain during the procedure. A prospective randomized controlled trial was conducted with 80 patients scheduled for ESWL. Patients were randomized into two groups: the video group (*n* = 40) received video-animated information in addition to standard verbal and written information, while the non-video group (*n* = 40) received only standard information. Anxiety levels were assessed using the State-Trait Anxiety Inventory (STAI), and pain perception, satisfaction, and willingness to repeat the procedure were evaluated using the Visual Analog Scale (VAS). The video group showed a significant reduction in situational anxiety (STAI-S scores: 40.1 ± 3.7 to 35.3 ± 2.7, *p* < 0.001) compared to the non-video group (39.7 ± 4.2 to 38.5 ± 4.5, *p* = 0.106). The video group also reported higher satisfaction scores (8.8 ± 1.3 vs. 7.2 ± 2.0, *p* < 0.01) and greater willingness to repeat the procedure (5.6 ± 2.0 vs. 3.6 ± 1.9, *p* < 0.01). No significant difference was observed in VAS pain scores between the groups (4.9 ± 1.3 vs. 5.4 ± 1.6, *p* = 0.298). Video-animated information significantly reduces situational anxiety and improves patient satisfaction and willingness to undergo ESWL again, without significantly affecting perceived pain levels. These findings support the use of multimedia tools in patient education to enhance the ESWL experience.

## Introduction

As a first-line option for renal calculi, Extracorporeal Shock Wave Lithotripsy (ESWL) provides a minimally invasive approach compared to surgical techniques, reducing patient morbidity [1]. Despite its effectiveness, ESWL can be a source of significant anxiety for patients due to the unfamiliarity of the procedure, the sounds produced by the lithotripter, and the requirement to remain motionless during treatment [2, 3]. Patient anxiety is a critical factor that can influence both the experience and outcomes of medical procedures, as heightened anxiety levels may lead to increased perceived pain, reduced satisfaction, and reluctance to undergo similar treatments in the future.

Recently, there has been an increasing focus on non-pharmacological interventions to alleviate patient anxiety and improve procedural satisfaction [4–6]. Among these, multimedia educational tools, such as video-animated information, have gained prominence for their ability to enhance patient understanding and reduce pre-procedural anxiety. These tools provide a visual and auditory explanation of the procedure, helping patients better comprehend what to expect, thereby reducing uncertainty and fear. Studies in various medical fields, including urology, have demonstrated the efficacy of video-based interventions in reducing anxiety and improving patient outcomes [5, 7, 8].

This study aims to evaluate the impact of video-animated information on anxiety levels, procedural satisfaction, willingness to undergo ESWL again, and perceived pain during the procedure. By comparing patients who received video-animated information in addition to standard verbal and written information with those who received only standard information, we seek to determine whether multimedia tools can enhance the patient experience during ESWL. Delivering animated video-based information to individuals prior to the procedure may greatly improve their comprehension of their condition and the planned intervention. This, in turn, can lead to a significant reduction in situational anxiety, an increase in patient contentment and readiness for repeat procedures, and a potential decrease in perceived pain.

## Materials and methods

This prospective randomized controlled trial sought to assess the impact of animated video-based information given to patients prior to ESWL on anxiety levels, procedural satisfaction, readiness to undergo the treatment again, as well as perceived discomfort during the procedure. This prospective study was conducted from February to March 2023 at Sakarya University Education and Research Hospital, the largest tertiary care center in the region. Prior to the study, approval was secured from the Sakarya University Ethics Committee. Written informed consent was obtained from all participants at least 24 h before the procedure. This timeline ensured adequate time for patients to review the information, ask questions, and consider their participation without time pressure, aligning with international ethical standards for elective procedures like ESWL.

Individuals scheduled for ESWL treatment for kidney stones were randomly allocated to the study in a 1:1 ratio. Participants were randomized 1:1 to either the video or control group using a web-based randomization system with permuted blocks of size 4. The sequence was generated by the hospital’s clinical trials unit, and allocation was concealed from researchers enrolling patients. A study nurse, unaware of group assignments, implemented the interventions. The inclusion criteria were first-time ESWL recipients with radiopaque kidney stones measuring 5–20 mm in size. In addition, the study included adult patients aged ≥ 18 years without upper age restriction. The exclusion criteria included patients with kidney anomalies, those with prior Double-J stent placement, individuals unwilling to participate, patients with ureteropelvic junction stenosis, and those with severe hydronephrosis.

**Fig. 1:**
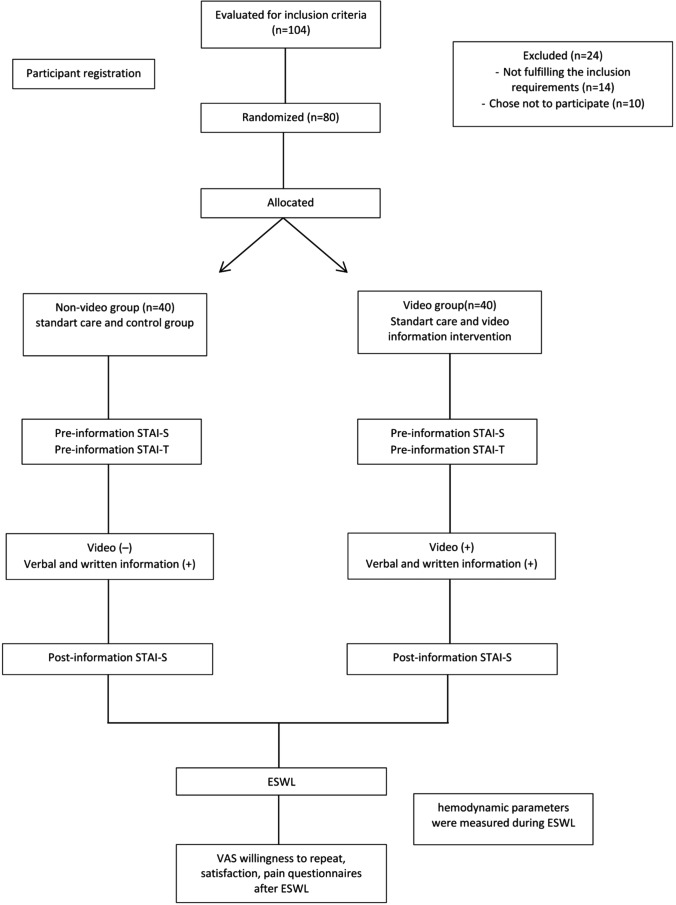
Diagram illustrating the process of the study

Before the patients started ESWL treatment, all participants’ age, gender, body mass index (BMI), State-Trait Anxiety Inventory (STAI) scores, stone location and diameters, and Hounsfield unit values ​​of the stones were recorded. STAI is an anxiety assessment questionnaire comprising 20 questions, and answers are taken on a 4-point Likert-type scale. STAI, a validated psychological assessment tool, was used to measure anxiety. Scores on the STAI range from 20 to 80, with higher values indicating greater anxiety levels [9]. The STAI consists of two subscales: state anxiety (STAI-S) and trait anxiety (STAI-T). STAI-T is a trait anxiety scale. It measures the general tendency of the person, i.e., the tendency to anxiety as a personality trait. STAI-S measures situation-specific anxiety. It evaluates the temporary anxiety level that the person feels at that moment [5]. All participants completed both the State-Trait Anxiety Inventory forms (STAI-T and STAI-S) before undergoing ESWL. Following this, animated video-based information was provided along with the standard verbal and written materials typically used for the video group. The non-video group received only the standard verbal and written materials. After completing the information process, STAI-S was applied to all participants again. Following the completion of the information process, ESWL was performed on the patients. Patient education (both verbal and video-assisted) was provided exclusively by a doctor who was not involved in performing ESWL procedures. All ESWL treatments were performed by one of three board-certified urologists blinded to group allocation. This separation of roles ensured consistency in information delivery and eliminated potential bias in treatment administration across both groups. Figure 1 presents the workflow of the study.

ESWL was performed using the ELMED Multimed EM lithotripter device (ELMED, Ankara, Turkey). The stone was localized and targeted using a C-arm fluoroscopy device, with the patient positioned on an electronic stretcher capable of movement in four directions and vertical adjustment within the ESWL suite. The procedure was initiated at an energy level of 1 kV and gradually increased to a maximum of 21 kV, depending on the patient’s tolerance. A pulse frequency of 80 shocks per minute was maintained, and a total of 2,500 pulses were delivered per patient. No analgesics were administered before or during the procedure. However, oral painkillers were prescribed to patients upon completion of the ESWL session to manage post-procedural discomfort.

VAS (Visual Analog Scale) is a widely used assessment tool that evaluates a person’s perceived condition, typically represented as a 10-unit straight line. One end of the line corresponds to “none” or the lowest level, while the other end represents “very severe” or the highest level. As the score approaches 10, the severity of the evaluated condition increases, whereas lower scores indicate a milder experience [4]. In our study, we adapted the VAS scale to assess willingness to repeat to the procedure, satisfaction, and pain. These evaluations took place after ESWL, and the outcomes were evaluated across the groups. In addition, the patients were monitored throughout the ESWL procedure. Hemodynamic parameters were measured and recorded, including mean systolic and diastolic blood pressures, heart rates, respiratory rates per minute, and oxygen saturation levels. These values were then compared between the groups to assess any differences in physiological responses during the procedure.

### Video information

The video-animated information was sourced from the patient information section of the EAU official website (https://patients.uroweb.org/videos/shock-wave-lithotripsy-swl-video/). This short, approximately 2.5-minute video explains the ESWL procedure using 3D animation, making it easier for patients to understand the process and post-procedure expectations. Since the video is in English, a doctor accompanied the patient during the viewing to provide real-time translation. The video-animated information was presented in the same room where written and verbal consent was obtained, allowing patients to rewind the video, ask the doctor questions about unclear points, and receive further explanations.

### Statistical analysis

Statistical analyses were performed using IBM SPSS Statistics version 22 (SPSS Inc., Chicago, IL, USA). The normality of data distribution was assessed using the Kolmogorov-Smirnov test. Continuous quantitative variables were presented as mean ± standard deviation (SD) for normally distributed data and non-normally distributed data; they were expressed with median and interquartile range. The independent samples t-test (Student’s t-test) was used for group comparisons of normally distributed variables, while the Mann-Whitney U test was applied for non-normally distributed variables. Categorical variables were summarized, and group comparisons were conducted using the chi-square test. A *p*-value below 0.05 was considered indicative of statistical significance.

The sample size for this study was calculated based on the effect sizes reported in a previous study by Kim, J. W. et al. [10], which investigated the effects of a heating pad on anxiety and pain during urodynamic studies. In their study, the State-Trait Anxiety Inventory (STAI) scores showed a mean difference of 7.1 points between the intervention and control groups, with a standard deviation (SD) of approximately 10. This corresponds to a Cohen’s d effect size of 0.71, indicating a medium-to-large effect. Using G*Power software (version 3.1.9.7) for an independent samples t-test with an alpha level of 0.05 and a power of 80%, a minimum of 34 participants per group (total 68 participants) was required to detect a similar effect size in our study. To account for potential drop-outs, we aimed to recruit 40 participants per group (total 80 participants). This sample size ensures adequate statistical power to evaluate the primary outcomes of anxiety (STAI) and pain (VAS) in our randomized prospective study.

## Results

104 participants were initially assessed for eligibility, of which 80 were randomly assigned to two groups (fourteen patients were excluded due to not meeting the inclusion criteria, and ten patients refused to take part in the study): the video group (*n* = 40) and the non-video group (*n* = 40). Figure 1 presents the workflow of the study. Table 1 presents the baseline demographic and clinical characteristics of the participants. No significant differences were observed between the two groups regarding age, gender, BMI, stone size, stone location, Hounsfield units, or pre-procedure STAI-S and STAI-T scores (*p* > 0.05 for all comparisons), suggesting that the groups were well-balanced at the start of the study. The homogeneous age distribution (mean 40.98 ± 8.2 vs. 41.23 ± 7.5 years) reflects both our inclusion criteria and our hospital’s typical demographics for first-time ESWL candidates. This age range represents the peak incidence of kidney stones in our region.

**Table 1:** Comparison of individual characteristics between groups

	Video group(*n* = 40)	Non-video group(*n* = 40)	*p*
Age (years) Gender (m/f) BMI (kg/m^2^) (mean ± SD)	40.98 ± 8.2 29/11 27.1 ± 2.2	41.23 ± 7.5 25/15 26.3 ± 2.2	0.888 0.340 0.137
STAI-T (mean + SD)	33.9 ± 3.9	33.1 ± 3.4	0.348
STAI-S (mean + SD)(pre-information)	40.1 ± 3.7	39.7 ± 4.2	0.695
Stone size (mm)(Mean ± SD)	10.7 ± 2.3	10.9 ± 1.9	0.684
Stone size (mm)(Median [IQR])	10.1 [9.3–10.5]	10.1 [9.3–10.6]	0.840
Stone side (Left/Right)	14/26	17/23	0.491
Hounsfield unit	772.2 ± 106.6	789.1 ± 100.4	0.468
Stone locations			0.731
Upper calyx	11	7	
Middle calyx	5	6	
Lower calyx	6	8	
Renal pelvis	18	19	

SD: standard deviation

IQR: interquartile range

The changes in STAI-S scores before and after the information intervention are presented in Table 2. In the video group, the mean STAI-S score decreased significantly from 40.1 ± 3.7 to 35.3 ± 2.7 (*p* < 0.001). Conversely, the non-video group demonstrated no notable change in STAI-S scores. (pre-information: 39.7 ± 4.2; post-information: 38.5 ± 4.5; *p* = 0.106). This suggests that the video-based information intervention was effective in reducing anxiety levels among patients.

**Table 2:** Comparison of changes in STAI-S scores between groups following the information provided

	Pre-information	Post-information	*P*
	STAI-S (mean + SD)	STAI-S (mean + SD)	
Video group	40.1 ± 3.7	35.3 ± 2.7	< 0.001
Non-video group	39.7 ± 4.2	38.5 ± 4.5	0.106

SD: standard deviation

STAI-S: State-Trait Anxiety Inventory, state anxiety

Table 3 presents a comparison of the hemodynamic parameters measured during the ESWL. The video group had significantly lower systolic blood pressure (140.7 ± 5.7 mmHg vs. 144.4 ± 7.3 mmHg, *p* = 0.014) and diastolic blood pressure (83.6 ± 4.7 mmHg vs. 86.5 ± 4.6 mmHg, *p* = 0.007) compared to the non-video group. No notable differences were found between the two groups in terms of respiratory rate, heart rate, or oxygen saturation (*p* > 0.05 for all).

**Table 3:** Comparisons of hemodynamic parameters measured during the ESWL across the groups

	Video group	Non-video group	*p*
Systolic pressure (mean ± SD)	140.7 ± 5.7	144.4 ± 7.3	0.014
Diastolic pressure (mean ± SD)	83.6 ± 4.7	86.5 ± 4.6	0.007
Respiratory rate (mean ± SD)	20.5 ± 3.8	21.7 ± 3.5	0.128
Heart rate (mean ± SD) Oxygen saturation (mean ± SD)	84 ± 8.7 98.3 ± 0.8	87.5 ± 9.7 98.2 ± 0.6	0.067 0.883

SD: standard deviation

Table 4 presents the post-procedure outcomes, including willingness to repeat the procedure, satisfaction, and pain. While there was no significant difference in VAS pain scores between the video group (4.9 ± 1.3) and the non-video group (5.4 ± 1.6, *p* = 0.298), the video group reported significantly higher satisfaction scores (8.8 ± 1.3 vs. 7.2 ± 2.0, *p* < 0.01) and greater willingness to repeat the procedure (5.6 ± 2.0 vs. 3.6 ± 1.9, *p* < 0.01).

**Table 4:** Comparison of willingness, satisfaction, pain across the groups

	Video group	Non-video group	*p*
VAS-pain (0–10) (mean ± SD)	4.9 ± 1.3	5.4 ± 1.6	0.298
VAS-satisfaction (0–10) (mean ± SD)	8.8 ± 1.3	7.2 ± 2	< 0.01
VAS-willingness to repeat (0–10) (mean ± SD)	5.6 ± 2	3.6 ± 1.9	< 0.01

SD: standard deviation

VAS: visual analog scale

## Discussion

ESWL, a commonly used non-surgical treatment for kidney stones in daily urology practice, can be a source of concern for patients. In particular, remaining motionless during the procedure, the sounds produced by ESWL devices, and a lack of full comprehension of the process contribute to increased patient anxiety. This prospective randomized controlled study sought to evaluate the impact of video-animated information on anxiety levels, procedural satisfaction, willingness to repeat ESWL, and perceived pain during the procedure. The findings demonstrate that the video-based intervention significantly reduced situational anxiety (STAI-S scores) and improved patient willingness to repeat the procedure and satisfaction, although it did not significantly affect perceived pain levels. These results highlight the potential of multimedia patient education tools in enhancing the psychological preparedness and overall experience of patients undergoing ESWL.

In contemporary urological practice, non-pharmacological methods are increasingly employed to alleviate patient anxiety and improve procedural satisfaction during various interventions, such as transrectal biopsy, urodynamic studies, and cystoscopy [8, 11, 12]. These methods include distraction techniques, such as listening to music, the sound of water, or watching videos using virtual reality (VR) glasses during the procedure [4, 13, 14]. Additionally, some clinicians utilize pre-procedural educational tools, such as informational videos, to prepare patients and reduce anxiety [7].

EAU patient information videos have gained significant popularity in recent years and are widely used to enhance patient understanding, reduce anxiety, and improve satisfaction before procedures. For instance, in a prospective randomized controlled trial, Bozkurt et al. [7] demonstrated that providing video-animated patient information before percutaneous nephrolithotomy (PCNL) surgery significantly reduced patient anxiety and positively impacted patient experience. Similarly, in another prospective randomized controlled study, Can et al. [15] reported that video-animated educational materials provided before double-J stent removal significantly improved STAI scores. **I**n line with the results of our study, Karalar et al. [16] conducted a study in which they divided 60 patients undergoing flexible ureteroscopy into two groups, providing video-animated information to one group while withholding it from the other. They observed a significant improvement in pre-procedural STAI scores in the group that received video-animated information.

Our study found a significant reduction in STAI scores in the group that received video-animated information before the ESWL. These findings align with previous studies, reinforcing the notion that video-animated information is more effective than traditional verbal or written methods in reducing pre-procedural anxiety. Despite no notable differences in pain levels measured by the VAS between the two groups, the video group reported higher satisfaction and greater willingness to repeat the procedure. This discrepancy may be attributed to the fact that pain perception is influenced by multiple factors, including individual pain thresholds and procedural factors, which may not be directly mitigated by informational interventions alone. However, the improved willingness to repeat the procedure and satisfaction suggest that the video intervention enhanced patients’ overall experience, likely by fostering a sense of control and reducing procedural apprehension.

The findings of this study offer valuable clinical insights. ESWL is a commonly performed procedure, and patient anxiety can negatively impact both the experience and outcomes. The use of video-animated information as a supplementary tool can be a simple, cost-effective, and scalable strategy to improve patient preparedness and satisfaction. This method is in line with the increasing focus on patient-centered care, which emphasizes the importance of recognizing and responding to the psychological needs of patients, which are integral to optimizing healthcare delivery.

However, this study has certain limitations. Firstly, the sample size, though adequately powered, was relatively small and limited to a single center, which could impact the ability to generalize the findings. Secondly, the study did not evaluate the long-term effects, such as the lasting reduction in anxiety or its influence on future medical procedures. Thirdly, the video-animated information was presented in English with real-time translation, which may introduce variability in comprehension among non-English-speaking patients. Fourthly, the study was not blinded. Future studies could explore the use of native-language videos and evaluate the long-term psychological and physiological effects of such interventions. In addition, this study did not record exact education durations, as the focus was on content standardization rather than time differences. However, the fixed video length and scripted verbal content likely minimized variability. Future studies could quantify time investments more precisely.

While our consent process (≥ 24 hours pre-procedure) complies with national regulations, we acknowledge that some jurisdictions mandate longer intervals (e.g., 48–72 hours) for elective treatments. Future multicenter studies should standardize consent timelines to accommodate diverse ethical requirements. However, our ≥ 24-hour consent window aligns with the Declaration of Helsinki’s principle that participants must have ‘adequate time’ for decision-making [17].

In conclusion, this study demonstrates that video-animated information significantly reduces situational anxiety, improves patient satisfaction, and increases willingness to undergo ESWL again without significantly affecting perceived pain levels. These findings support the integration of video-animated patient information tools into routine clinical practice to enhance patient experience and outcomes. Future research should optimize these interventions and explore their applicability in other medical procedures.

## Data Availability

No datasets were generated or analysed during the current study.
